# Brain cholesterol therapy for Huntington's disease – Does it make sense?

**DOI:** 10.1002/ctm2.1746

**Published:** 2024-06-25

**Authors:** Elena Cattaneo, Roger A Barker

**Affiliations:** ^1^ Department of Biosciences University of Milan Milan Italy; ^2^ Istituto Nazionale di Genetica Molecolare ‘Romeo ed Enrica Invernizzi’ Milan Italy; ^3^ Cambridge Stem Cell Institute and John van Geest Centre for Brain Repair Department of Clinical Neuroscience University of Cambridge Cambridge UK

1

The use of cholesterol as a therapy for Huntington's disease (HD) might seem peculiar at first, but it has a strong experimental foundation. HD, like other chronic neurodegenerative disorders, lacks disease‐modifying therapies and has limited symptomatic treatments for the movement and psychiatric problems that characterise it. The additional abnormal sleep patterns and weight loss are managed with supportive approaches. These disturbances arise from a CAG expansion in the huntingtin gene, leading to dysfunction and death of neurons in the cortex and striatum. The disease process likely begins years before clinical presentation. Work is ongoing to identify the earliest pathology stages using imaging, clinical and biomarker measures.^1^

One‐fifth of all cholesterol in the human body is in the brain, where it is crucial for neuronal function and membrane integrity. The blood–brain barrier (BBB) prevents the uptake of lipoprotein‐bound cholesterol from the systemic circulation; thus, brain cholesterol originates from local synthesis, primarily in astrocytes, through a multistep pathway involving several enzymes. Neurons have limited cholesterol biosynthesis capabilities and rely on astrocytes for their cholesterol supply, so as to protect them from the potentially harmful effects of excessive cholesterol production. Over the last 25 years, work in the Cattaneo lab has demonstrated across a wide variety of models that cholesterol biosynthesis is severely reduced in the HD brain.^2^ A meta‐analysis of all the cognitive data from studies on HD mice exposed to cholesterol‐increasing strategies in the brain also showed that, regardless of the approach type, delivery system, mouse genotype and time of administration, the cognitive performance of treated HD mice greatly improved relative to untreated HD groups.^2^, ^3^ As such, the concept of increasing cholesterol levels or stimulating its biosynthesis in the HD brain is well justified.

Various strategies have been explored to raise cholesterol levels in the HD mouse brain.^2^ Direct infusion into the brain of 369 µg of cholesterol via an osmotic‐minipump rescued both motor and cognitive abnormalities, while 15 µg improved cognition only. A gene therapy approach forcing expression of the SREBP2 gene (the transcription factor promoting cholesterol biosynthesis) in astrocytes, again, leads to a rescue of cognitive and motor functions in HD mice. Intraperitoneal injection of cholesterol‐loaded nanoparticles, modified to cross the BBB, was found to be successful in two HD mouse models, providing long‐lasting behavioural and neuropathological benefits with no side effects.^3^ Indeed, even giving it ahead of overt clinical features slowed disease onset. More recently, an intranasal delivery approach has shown great potential in mouse models of HD.^2^

A trial to restore cholesterol biosynthesis in HD patients with AB‐1001 gene therapy has started but paused until 2025.^4^ Formerly known as hCYP46A1, this gene converts approximately 6 mg of brain cholesterol per day into its hydrophilic catabolite, 24‐hydroxycholesterol (24OHC), which then leaves the brain and is detectable in plasma. This therapy has been primarily designed for Alzheimer's disease (AD), where cholesterol accumulates pathologically and contributes to aggregate formation, while in HD the reverse situation is seen.

But what is the evidence of changes in cholesterol biosynthesis in the human HD brain? Measurements of 24OHC in plasma from HD patients have thus far yielded inconclusive results, and longitudinal studies are still awaited. The recently developed PET tracer that binds to CYP46A1 will be very informative, as it will allow for the visualisation of cholesterol metabolism with spatial resolution in the living human brain.^5^ Studies investigating the effects of statins on disease progression in HD patients may also be informative, but they are complicated by the fact that these drugs have multiple actions and primarily target peripheral cholesterol, although many can pass the BBB. Animal data suggest that statins should worsen HD. Initial studies in humans show less severe disease in the statin‐treated patients, but these studies are unable to distinguish between the effects that statins have on lowering the lipid level from the fact that the underlying hyperlipidemia itself may be directly slowing down disease progression.^6^

At this stage, the strongest evidence supporting a cholesterol trial in HD patients comes from the animal studies. However, translating this data into human trials presents challenges, including a lack of intellectual property and resistance to using cholesterol as a drug. Optimal dosage, efficacy, and biosafety of GMP‐grade cholesterol administration needs investigation, as does how to best deliver it to the brain. However, neither of these challenges seem insurmountable as for example, subcutaneous implantation of minipumps have already been employed in humans for direct brain delivery of treatments for glioma and Parkinson's Disease. Inorganic, organic (liposomal), or hybrid nanoparticles have been used to deliver chemotherapeutic agents for brain tumours.^7^ Intranasal delivery is another viable method that has been trialled in AD patients already.^8^

Moreover, assessing the efficacy of cholesterol therapy in HD requires reliable biomarkers and robust outcome measures. The failure of previous HD trials, such as those involving antisense oligonucleotides, underscores the importance of having clinically relevant endpoints that accurately reflect disease progression and treatment response at the right site.^9^ Looking at multiple measures, including clinical assessments, neuroimaging, and biomarker analyses of blood/CSF markers such as neurofilament‐light‐chain, GFAP and even mutant huntingtin, may offer a more comprehensive evaluation of therapeutic outcomes in cholesterol therapy trials for HD. As none of these are ideal, using a combination of measures may be more useful approach.^10^ Finally, HD has a prolonged prodromal phase during which neurons are dysfunctional but not yet lost. Providing cholesterol during this period may help prolong neuronal and synaptic functionality.

However, trialling such approaches in premanifest patients raises a number of ethical (around safety in essentially normal individuals) and practical measures, around reliably tracking the disease process in the absence of clinical signs. As such, a first in human study is likely to be an open label dose finding trial in patients with early manifest disease, looking at tolerability and feasibility but also with exploratory end points around efficacy and ideally target engagement.

In conclusion, exploring cholesterol in trials for HD has a clear rationale and evidence base, and so the time is now right to take this to early phase clinical trials.
